# An Introduction to Dermatology

**Published:** 1900-06

**Authors:** 


**An Introduction to Dermatology.** By NORMAN WALKER, M.D. Pp. xvi., 247. Bristol: John Wright and Co. 1899.

Upon the whole an excellent work, well up-to-date, with a modest title. There are thirty coloured photogravure plates and thirty-four drawings. Some of the coloured plates are rather inartistic; but, with few marked exceptions, this condition seems almost inseparable from medical drawings. The plates have very little margin, but even this is better than having larger separate ones. Plate xxvi. seems to us the best, and the quality of the paper is better—smoother and not quite so "hog-skinned." We confess we do not like engravings from photos; the details, when magnified, compare very indifferently with true photographs, but these latter must doubtless have increased the cost of the work. The index is in clear, good print, full, and very free from mistakes. The text of the work is good, and contains few errors; but surely "numular" (p. 182) should read "nummular"; and on p. 16, sixth line from bottom, ʒviij. is evidently meant for ℥viij. Some of the remarks on diagnosis are pertinent: thus, "dermatology is not practical chemistry." The value of the thermometer is well stated.

That comprehensive subject, eczema, is cleverly dealt with, and the details of treatment, we hold, are about the best and the most sensible we have ever come across. On p. 183, speaking of the late manifestations of syphilis, we can thoroughly endorse the author's views, and also that "a small pigmented scar in the neighbourhood of the knee will clear up the nature of a very obscure eruption"; but we will go further, and say that the scar may even have lost its pigment and yet furnish most valuable aid.

We cannot understand the rationale for placing pediculosis under "hemorrhages"—the very slight bleeding seems to us to about measure the reason for doing so! Nor can we admit seborrhœa as a form of psoriasis—probably they are both parasitic, but this need not involve unity. However, the author is such a conscientious worker that we feel sure when he sees reason to alter his views he will do so; and as a second edition will certainly be called for, there will be an opportunity of correcting the more trivial errors we have pointed out.

